# Contrasting diversity patterns of prokaryotes and protists over time and depth at the San-Pedro Ocean Time series

**DOI:** 10.1038/s43705-022-00121-8

**Published:** 2022-04-13

**Authors:** Yi-Chun Yeh, Jed A. Fuhrman

**Affiliations:** Department of Biological Sciences, University of Southern California, Los Angeles, CA 90089-0371 USA

**Keywords:** Biodiversity, Microbial ecology

## Abstract

Community dynamics are central in microbial ecology, yet we lack studies comparing diversity patterns among marine protists and prokaryotes over depth and multiple years. Here, we characterized microbes at the San-Pedro Ocean Time series (2005–2018), using SSU rRNA gene sequencing from two size fractions (0.2–1 and 1–80 μm), with a universal primer set that amplifies from both prokaryotes and eukaryotes, allowing direct comparisons of diversity patterns in a single set of analyses. The 16S + 18S rRNA gene composition in the small size fraction was mostly prokaryotic (>92%) as expected, but the large size fraction unexpectedly contained 46–93% prokaryotic 16S rRNA genes. Prokaryotes and protists showed opposite vertical diversity patterns; prokaryotic diversity peaked at mid-depth, protistan diversity at the surface. Temporal beta-diversity patterns indicated prokaryote communities were much more stable than protists. Although the prokaryotic communities changed monthly, the average community stayed remarkably steady over 14 years, showing high resilience. Additionally, particle-associated prokaryotes were more diverse than smaller free-living ones, especially at deeper depths, contributed unexpectedly by abundant and diverse SAR11 clade II. Eukaryotic diversity was strongly correlated with the diversity of particle-associated prokaryotes but not free-living ones, reflecting that physical associations result in the strongest interactions, including symbioses, parasitism, and decomposer relationships.

## Introduction

Marine microbial communities consist of all three domains of life: Bacteria, Archaea, and Eukaryota. Together, these organisms perform a wide range of marine biogeochemical process as they represent key trophic roles in microbial food webs. However, only a few studies have thoroughly surveyed all these components together [1, 2]. The lack of comprehensive investigations is due, in part, to the difficulties of accessing the diversity across three domains. The emergence of high-throughput sequencing techniques allows us to identify the community composition using appropriate marker genes (e.g., SSU rRNA genes). Recent studies have shown that a universal primer set that amplifies both prokaryotes and eukaryotes can quantitatively survey the whole microbial community in a single PCR reaction [3–6], allowing direct comparisons with a single denominator.

Understanding the natural variability of community dynamics relies on long-term observations. The best-known microbial ocean time series programs include the Hawaii Ocean Time series (HOT), the Bermuda Atlantic Time Series, both far offshore open ocean systems, the Blanes Bay Microbial Observatory nearshore in the NW Mediterranean, and the San-Pedro Ocean Time series (SPOT) [7–10]. SPOT is about 20 km off the coast of Southern California, in a coastal basin with about 900 m water depth and restricted horizontal advection below about 500 m [11], resulting in a persistent hypoxic environment at depth. Thus, SPOT provides a good opportunity to examine the spatiotemporal variation of microbial communities in a subtropical mesotrophic marine system.

Here, we present a 14-year study that uses SSU rRNA sequencing with a universal primer set (515Y/926R) to investigate the spatiotemporal variation of the marine microbial community from two size fractions at SPOT. The 16S and 18S sequences were denoised into amplicon sequence variants (ASVs) using DADA2 implemented in QIIME2 [12], which allows resolutions down to single-nucleotide differences. The 515Y/926R primer set has been widely used for prokaryotic community analysis [13–15]. However, only a few studies have attempted to use it as a universal primer set, even though it has better practical eukaryotic coverage compared to most eukaryote-specific primers [16] and eukaryotic amplicons are always present. This study is the first to use its universal nature to survey a microbial long-term time series. With a previous published methodology, we were able to simultaneously survey prokaryotic 16S, chloroplast 16S (representing phototrophic eukaryotes), and 18S rRNA (representing phototrophic and heterotrophic protists) in a single PCR reaction. The prokaryotic 16S rRNA communities from two size fractions allow us to further separate free-living prokaryotes (0.2–1 μm) from larger ones and those attached to or associated with particles (1–80 μm).

The ability to simultaneously survey prokaryotes and protists allows us to determine the extent that diversity patterns and spatiotemporal stabilities depend on functional types or traits. Prokaryotes and protists exhibit differences in body size, metabolic capacity, population size, trophic characteristics, maximum growth rates, and dispersal potential, which may influence their community dynamics along spatial and temporal gradients. Previous studies have shown that eukaryotic communities were driven more strongly by environmental filtering relative to dispersal processes than were prokaryotic communities [17–19]. These findings indicate that the fundamental differences between prokaryotes and protists may determine the mechanisms structuring the community composition at spatial scales. However, prior studies generally provide only snapshot or short-term views of community dynamics, incapable of indicating differences in characteristics like long-term stability. Thus, our goals were to (1) examine the extent these different taxonomic/functional groups experience different temporal and vertical dynamics in alpha- and beta-diversity, (2) identify the taxa associated with the diversity patterns, and (3) examine relationships among diversity patterns that point to potential interactions between the groups.

## Results

### Environmental conditions

**Fig. 1:**
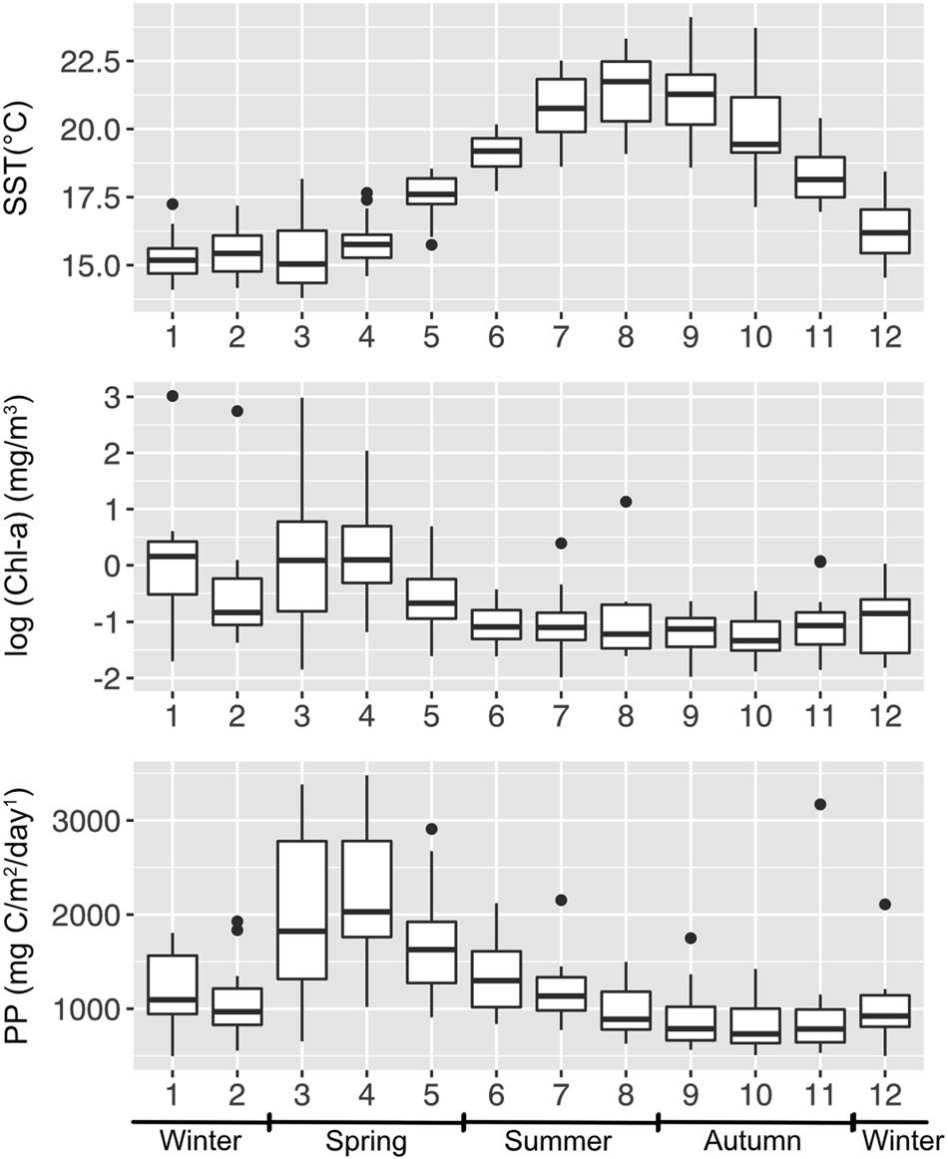
Monthly average sea surface temperature (SST), satellite chlorophyll-a concentration (Chl-a), and satellite primary productivity (PP) at the SPOT location during 2005–2018. The black lines within the box plots represent the median values, and the box bottom and top show the 25th and 75th percentile. The whiskers represent the lower and the upper bounds, and the dots represent outliers.

Satellite data shows that SPOT has a seasonal cycle of chlorophyll and primary production (Fig. 1). The highest monthly chlorophyll-a concentration and primary productivity generally occurred in March–May, while the minima occurred in September-November. Thus, the seasons were defined here with March-May as spring, June–August as summer, September-November as autumn, and December-February as winter. The whole water column has strong vertical environmental gradients, characterized by principal component analysis on environmental variables, including temperature, dissolved oxygen, fluorescence, NO_2_ + NO_3_, and PO_4_. The first principal component was positively correlated with nutrients, and negatively correlated with temperature and dissolved oxygen, generally ordinating the cold and nutrient-rich deeper samples in the positive range and warm and nutrient-depleted surface samples in the negative range (Fig. S1).

### Microbial community composition

**Fig. 2:**
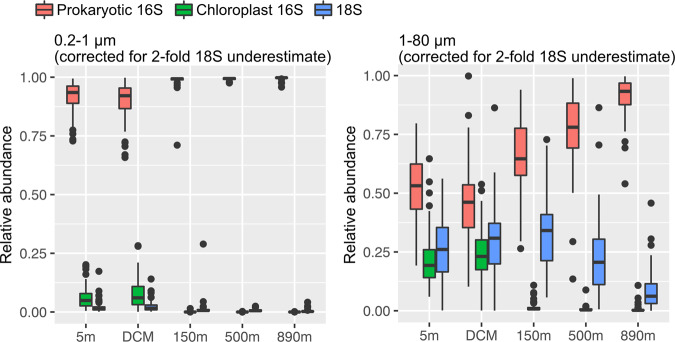
Dominance of prokaryotes in both size fractions, as shown by the proportions of prokaryotic 16S, chloroplast 16S (representing phototrophic eukaryotes), and 18S reads (including Metazoa sequences) found in 0.2–1 μm and 1–80 μm size fractions. The corrected values include a twofold adjustment of the 18S sequences to account for length-based bias in sequencing, determined from mixed mock communities (see text, and uncorrected data in Fig. S3).

A total of 908 samples collected at the SPOT location during 2005–2018 were analyzed by SSU rRNA sequencing with the universal primers (515Y/926R) that amplify prokaryotic 16S, chloroplast 16S (representing phototrophic eukaryotes), and 18S rRNA genes simultaneously. After quality control filtering, merging, chimera removal, and sequencing bias correction [3], the 0.2–1 μm size fraction sequence data was partitioned into an average of 92–100% prokaryotic 16S, 5–6% chloroplast 16S, and <2% eukaryotic 18S. The 1–80 μm size fraction sequences were partitioned into 46–93% prokaryotic 16S, 19–23% chloroplast 16S, and 6–34% eukaryotic 18S (Fig. 2). Microbial community composition in the 1–80 μm size fraction shifted from communities that were roughly evenly distributed into these three categories at the surface to prokaryote-dominated communities at depth. So as expected, the 0.2–1 μm size fraction mainly captured prokaryotes, with picoeukaryotic chloroplast and 18S sequences averaging <5% (Fig. 2). The 1–80 μm size fraction collected all three categories, yet the larger fraction was interestingly still dominated by prokaryotes, averaging >50% at all depths. Also, because the euphotic zone at SPOT is generally shallower than 100 m, with the DCM typically averaging 5 to 66 m, we expected significant contributions from chloroplast 16SS (phototrophic eukaryotes) only in the 5 m and DCM samples. However, there was still a substantial contribution at 150 m, ranging 0.15–10.8%, which are primarily sinking diatoms (Fig. S8).

### Prokaryotic community composition

**Fig. 3:**
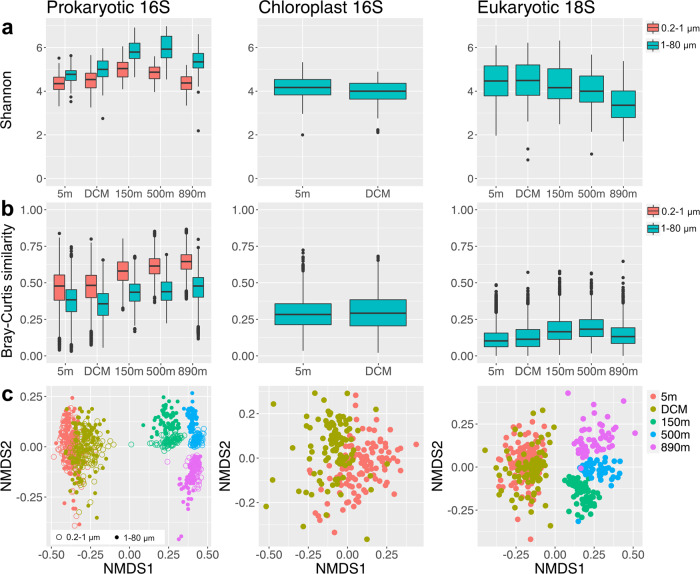
Alpha- and beta-diversity patterns for prokaryotic 16S, chloroplast 16S, eukaryotic 18S (excluding Metazoa sequences). **a** Shannon (H′) index, showing a diversity maximum at 150 and 500 m for prokaryotes and at 5 m and DCM for eukaryotes. **b** Patterns of pairwise Bray-Curtis similarity between all sampling dates within each size fractions for each sampling depth, showing that temporal stability of prokaryotes increased with depth (i.e., higher average similarities between all sample pairs), and that eukaryote communities were less temporally stable than prokaryotes. **c** Nonmetric multidimensional scaling (NMDS), with ordination computed based on Bray–Cutis distance of prokaryotic 16S, chloroplast 16S, and eukaryotic 18S communities, showing depth stratification for all three types, and that for prokaryotes there was a greater differentiation between size fractions in the depths ≥150 m.

**Fig. 4:**
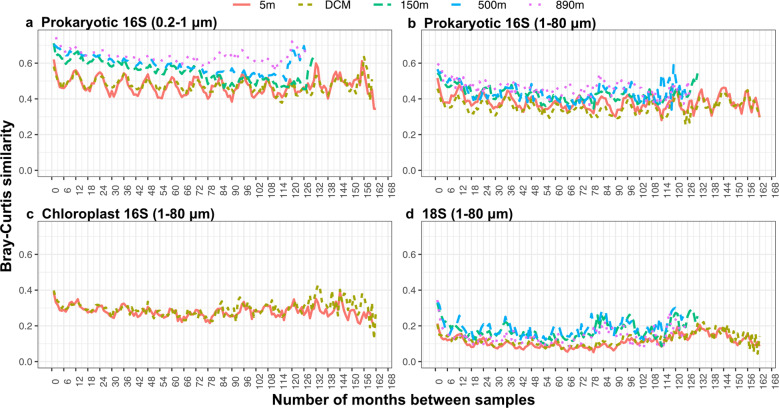
Temporal patterns in community similarity of free-living prokaryotic 16S (0.2–1 μm), particle-associated or larger prokaryotic 16S (1–80 μm), chloroplast 16S and 18S (excluding Metazoa sequences) communities. Free-living and particle-associated or larger prokaryotes at 5 m and the DCM exhibited a clear annual recurrence pattern, with peaks in similarity at 12-month intervals (i.e. comparing all samples 12, 24, 36, etc. months apart) and lowest similarity in opposite seasons, i.e. 6, 18, 30 etc. months apart. Note that as number of years increased between samples, the 5 m depth free-living prokaryote similarities oscillated fairly steadily around an average of ~0.5, suggesting variability within a steady range of community compositions, while the 150 and 500 m free-living prokaryotes showed a general decline in similarity from about 0.7 at short intervals to about 0.5 at longer intervals, suggesting a tighter range of compositional change particularly for samples collected within a few years of each other. The 890 m similarities are more persistently high across all intervals.

We examined free-living (0.2–1 μm) and particle-associated or larger (1–80 μm) prokaryotic communities using prokaryotic 16S rRNA sequences. The Shannon index values of particle-associated or larger prokaryotic communities were significantly higher than free-living prokaryotic communities (Fig. 3a, Kruskal–Wallis test, *p* < 0.001). The greatest Shannon index values were observed in the large size fraction at 150 m and 500 m (Dunn’s test, *p* < 0.001). Pairwise Bray–Curtis similarity was used to assess temporal stability (variation in community composition over time) within each sample category, and there was a trend indicating that temporal stability increased with depth in both size fractions, shown by higher average similarity (Fig. 3b). Nonmetric multidimensional scaling (NMDS) analysis shows that prokaryotic community structure was clustered by sampling depth, with a clear separation between euphotic zone samples (5 m and DCM) and deeper samples (>150 m) (Fig. 3c). The effect of sampling depth was highly significant explaining 48% of ASV composition, against 5% attributed to the size fraction, and 2% attributed to season (Table 1). The effects of size fraction and season were more evident when the dataset was partitioned by depth (Table 2). Seasonal effects significantly explained 14% and 11% of variability at 5 m and DCM respectively, whereas seasonal changes were not as prominent below the euphotic zone, explaining only <6% of the variability. Annually reoccurring patterns were also observed at 5 m and the DCM when the interval in months between samples was plotted against Bray-Curtis similarity (Fig. 4a, b), as shown by peaks in similarity when samples on the same or similar calendar month were compared, whether 1, 2, 3, or more years apart, and lowest similarities when “opposite” months were compared (i.e., separated by 6, 18, 30, etc. months). The effects of size fraction, on the other hand, were more important below the euphotic zone, explaining 29–36% of the variability (Table 2).

**Table 1:** Sources of overall variation, by PERMANOVA test, of the prokaryotic 16S, chloroplast 16S, and eukaryotic 18S (excluding Metazoa sequences), considering all depth and dates together in a single analysis.

	*R*^2^ (Depth)	*R*^2^ (Season)	*R*^2^ (Size fraction)
Prokaryotic 16S	0.48	0.02	0.05
Chloroplast 16S	0.07	0.06	
Eukaryotic 18S	0.18	0.02	

The *R*^2^ values represent the fraction of overall variation ascribed to depth, season, and size fraction. Note that chloroplasts were only evaluated at 5 m and DCM depths, and chloroplast 16S and eukaryote 18S were only evaluated in the large size fraction. The seasons were defined here with March–May as spring, June–August as summer, September–November as autumn, and December–February as winter. The *p* values were all 0.001.

**Table 2:** Sources of overall variation, by PERMANOVA test, of the prokaryotic 16S, chloroplast 16S, and eukaryotic 18S (excluding Metazoa sequences), considering each depth separately.

Depth group	Prokaryotic 16S				Chloroplast 16S		Eukaryotic 18S	
	Season		Size fraction		Season		Season	
	*R*^2^	*p* value	*R*^2^	*p* value	*R*^2^	*p* value	R^2^	*p* value
5 m	0.14	0.001	0.13	0.001	0.10	0.001	0.07	0.001
DCM	0.11	0.001	0.14	0.001	0.10	0.001	0.06	0.001
150 m	0.06	0.001	0.29	0.001			0.09	0.001
500 m	0.03	0.002	0.36	0.001			0.14	0.001
890 m	0.03	0.003	0.36	0.001			0.09	0.001

The *R*^2^ values represent the fraction of overall variation ascribed to season and size fraction for each depth. For prokaryotic 16S, season and size fraction contribute similarly to variation in the 5 m and DCM samples, but for samples ≥150 m, the variation due to size fraction is about 2–3× stronger than it is for season, and 2–3× stronger than the euphotic zone seasonal effects. For chloroplast 16S in the large size fraction, season explained the same amount of variation at 5 m and DCM. For eukaryotic 18S, seasonal variation at 500 m is about 2× strong than the other depths. Note that chloroplasts were only evaluated at 5 m and DCM depths, and chloroplast 16S and eukaryote 18S were only evaluated in the large size fraction.

**Fig. 5:**
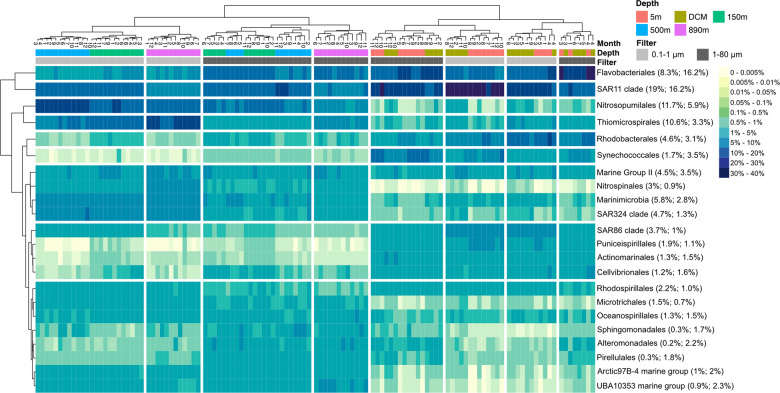
Partitioning of major taxonomic groups by depth, size fraction, and month of the year shows strong stratification and seasonal effects. Heatmap of monthly average prokaryotic 16S communities at the order level (only dominant orders were selected, if their mean relative abundance is >1%). Columns were clustered based on Bray–Curtis distance. Rows were clustered based on Euclidean distance. The top row is colored by sampling depth, and second row indicates the size fraction. The dendrogram on top shows that prokaryotic communities primarily clustered by sampling depth (surface vs. depth) and then by size fraction. The numbers in parentheses show the overall average relative abundances in the 0.2–1 μm and 1–80 μm size fraction, respectively.

The taxonomic composition (Figs. 5 and S9) shows that, in general, SAR11 and Flavobacteriales were abundant in all depths and both size fractions. At 5 m and the DCM, SAR11 dominated in summer/autumn, whereas Flavobacteriales dominated in spring/winter. Rhodobacterales, SAR86, and Puniceispirillales (SAR116) predominated in the euphotic zone, mainly in the small size fraction, whereas Synechococcales, Actinomarinales, Cellvibrionales were abundant in the large size fraction in the euphotic zone. Additionally, Nitrosopumilales Archaea, Thiomicrospirales, Nitrospinales, Marinimicrobia, and SAR234 predominated at the deeper depths, mainly in the small size fraction. Although other major groups (such as Artic97B-4 marine groups, UBA10353 marine groups, and Sphingomonadales) did not show a clear pattern of distribution, they were generally present particularly in the large size fraction in deeper depths.

**Fig. 6:**
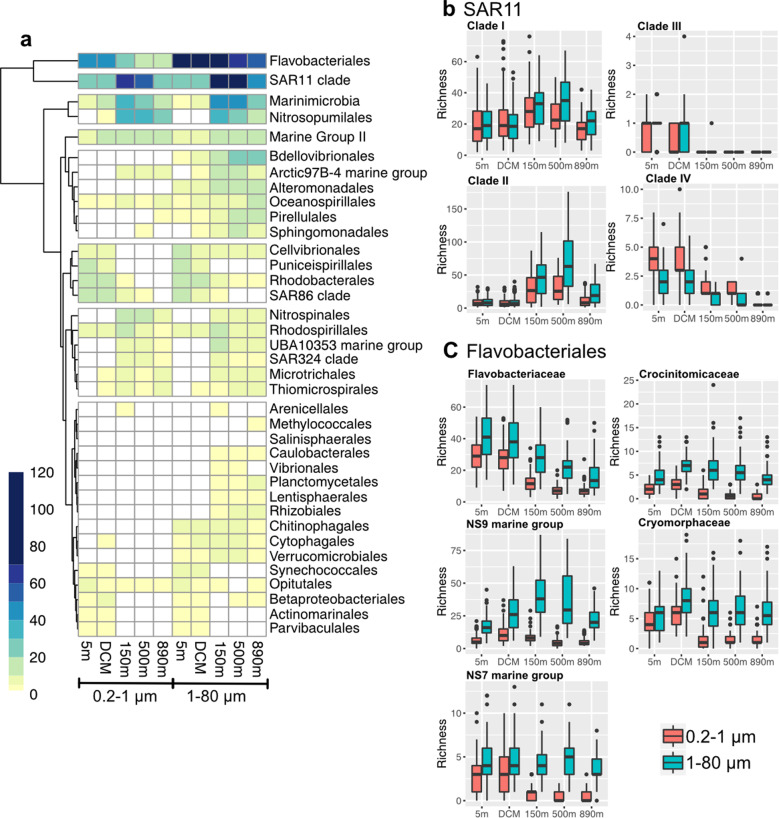
Diversity within major groups differs sharply between taxa, depth, and size fraction. **a** Heatmap of rarefied ASV richness observed within each prokaryotic order at each sampling depth (rows were clustered based on Euclidean distance). There were endemic taxonomic groups present at depth, such as Nitrospinales, UBA10353 marine groups, and SAR324. Note Flavobacteriales and SAR11 both exhibited high richness at 150 and 500 m, thus (**b**) and (**c**) were further analyzed for richness patterns among subclades. **b** SAR11 clade I was ubiquitously distributed in the water column, whereas SAR11 clade II showed high richness at 150 and 500 m. **c** Flavobacteriales subclades showed that all these groups were more diverse in the large size fraction. Among them, NS9 marine groups were high in richness at 150 and 500 m.

To examine the extent these endemic groups contributed to the high richness in the large size fraction at 150 and 500 m, the number of ASVs observed within each group was calculated (Fig. 6a). The heatmap shows that the richness at 150 and 500 m was not only contributed by the orders that were endemic to deeper waters, but also by SAR11 and Flavobacteriales. As SAR11 and Flavobacteriales have been found to have niche differentiation between major subgroups [20–25], the ASV richness within each subgroup was further compared (Fig. 6b, c). The results show that SAR11 clade I was present in high richness regardless the sampling depth and size fraction. In contrast, SAR11 clade II members were only found in high richness at 150 and 500 m. For Flavobacteriales, the Flavobacteriaceae, NS7, NS9, Crocinitomicaceae, and Crymorphaceae were all found in high richness in the larger size fraction. Among these subgroups, the NS9 had particularly high richness at 150 and 500 m.

### Phototrophic eukaryotic community composition

**Fig. 7:**
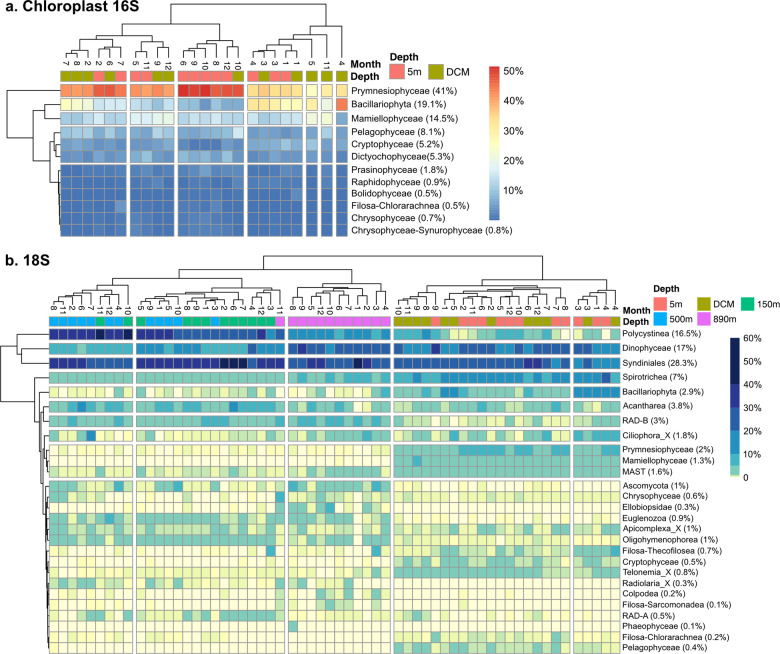
Relative abundance of major eukaryote groups varied with season and depth shown by monthly average chloroplast 16S (representing phototrophic eukaryotes) and 18S (excluding Metazoa sequences) communities in 1–80 μm size fraction. Only abundant classes were selected. i.e. with relative abundance of >2% in any sample. Columns were clustered based on Bray-Curtis distance. Rows were clustered based on Euclidean distance. **a** Chloroplast 16S communities were mostly dominated by Prymnesiophyceae in summer/autumn and Bacillariophyta (diatoms) in spring/winter. **b** 18S communities were dominated by Syndiniales throughout the water column, followed by dinoflagellates, rhizarians (polycystine radiolarians mostly deeper, acantharians at all depths), and ciliates (mostly top 3 depths). The numbers in parentheses show the overall average relative abundances.

The chloroplast 16S rRNA gene was used to identify phototrophic eukaryotic communities (excluding most dinoflagellates [4]); from the 1–80 μm size fraction in the euphotic zone (5 m and DCM). When comparing the alpha- and beta-diversity between sampling depths, the Shannon index values of chloroplast 16S communities at 5 m were slightly higher than that at DCM (Fig. 3a, Kruskal–Wallis test, *p* = 0.008). No significant differences in pairwise Bray–Curtis similarity of all samples within a given depth were found between 5 m and the DCM (Fig. 3b), which indicates these euphotic depths have similar overall phytoplankton community stability. NMDS and PERMANOVA show that samples were slightly clustered by sampling depth, explaining 7% of ASV composition (*p* < 0.001), against 6% attributed to season (Fig. 3c and Table 1). After partitioning by depth, the seasonal effect at 5 m and DCM both explained 10% of variability (Table 2). The major classes were Prymnesiophyceae (41%), Bacillariophyta (19%), Mamiellophyceae (15%), Pelagophyceae (8%), Dictyochophyceae (5%) and Cryptophyceae (5%). Prymnesiophyceae and Bacillariophyceae predominated in all samples with Prymnesiophyceae as the main representative during summer/autumn, and Bacillariophyceae dominated during spring/winter (Fig. 7a).

### Protistan community composition

18S rRNA gene was used to identify protistan communities from the 1–80 μm size fraction. Eukaryotic 18S communities were sporadically dominated by Metazoa, including Arthropoda (usually copepods), Cnidaria, Urochordata (usually larvaceans), Annelida, Ctenophora (Fig. S6). Considering adults of these groups are typically larger than 80 μm, these Metazoa sequences likely represented juveniles, eggs, or organismal fragments that we expect were sporadically captured in this size fraction (accounted for an average of 22% of 18S sequences). As we focused on protists here, we excluded Metazoan sequences in the following analyses. The Shannon index values of protistan communities were significantly different among sampling depths (Kruskal–Wallis test, *p* < 0.001). The Shannon index was lowest at 890 m and peaked at the DCM. NMDS and PERMANOVA tests show that samples were clustered by sampling depth, explaining 18% of ASV composition (*p* < 0.001), against 2% attributed to season (Fig. 3c and Table 1). When considering the seasonal effect by depth, season explained 14% of total variation at 500 m and 6–9% at 5 m, DCM, and 890 m (Table 2).

Protistan taxonomic composition was dominated by Syndiniales (28%), Dinophyceae (17%), Polycystinea (17%), Spirotrichea (7%), Acantharea (4%), RAD-B (3%), and Bacillariophyta (3%). Dinophyceae, Spirotrichea, Bacillariophyta, Prymnesiophyceae, Mamiellophyceae, and MAST were important contributors above euphotic zone, while Radiolaria (Polycystinea, Acantharea, and RAD-B) and Syndiniales increased in relative abundances below the euphotic zone (Fig. 7b). We also compared community composition of phototrophic eukaryotes (phytoplankton) as examined by two different markers, chloroplast 16S and nuclear 18S rRNA genes, and found broad general agreement between them for most subgroups (Figs. S10–S12).

### Diversity relationships among free-living and particle-associated prokaryotes, phototrophic eukaryotes, and protists

**Fig. 8:**
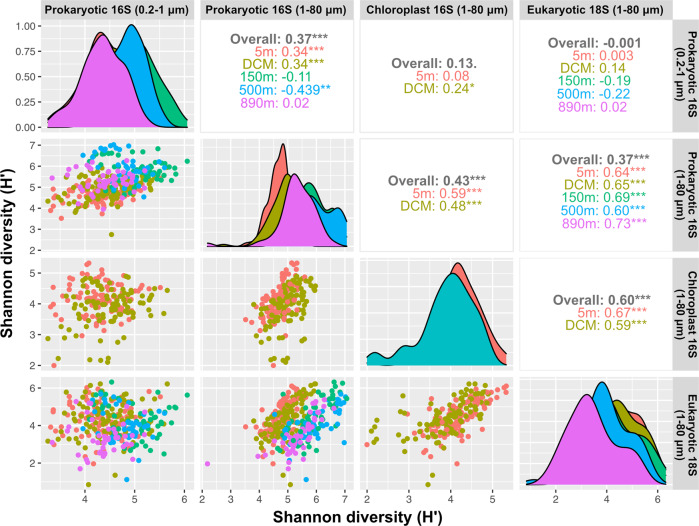
Relationships of diversity between microbial types at different depths. Scatterplots of Shannon diversity between each two communities are shown on the lower and left side. Pearson’s correlations considering all depths and depth-specific correlations are shown on the right. The distributions of Shannon diversity color coded by depth is shown on the diagonal. While many correlations were strong, there were notably weak correlations between diversity of eukaryotes (or chloroplasts) and free-living prokaryotes (0.2–1 μm). Significance levels (“.” *p* < 0.1; “*” *P* < 0.05; “**” *P* < 0.01; “***” *P* < 0.001).

When examining the relationships between diversity patterns of the different categories (Fig. 8), the strongest correlation was between overall protistan diversity and that of phototrophic eukaryotes via chloroplasts (Pearson’s correlation, *r* = 0.60, *P* < 0.001), unsurprising because one is a major subset of the other. Diversity of protists in general (18S) as well as phototrophic protists alone (chloroplasts) were both significantly correlated to that of particle-associated or larger prokaryotes (Pearson’s correlation *r* = 0.37 and 0.43, *P* < 0.001). In addition, diversities of free-living and particle-associated or larger prokaryotes were positively correlated with each other (Pearson’s correlation, *r* = 0.37, *P* < 0.001). The weakest correlations were between diversity of protists and free-living bacteria (r = ~0)

## Discussion

This is the first long-term study of the microbial community from two size fractions (0.2–1 and 1–80 μm) using universal primers that allow direct quantitative comparisons among prokaryotic 16S, chloroplast 16S (phototrophic eukaryotes), and 18S all at the same time. When comparing the proportions of prokaryotic 16S, chloroplast 16S and 18S rRNA genes obtained from the two size fractions, our results indicate that even though the smaller size fraction is often considered the “prokaryotic” one, when examined on a gene-by-gene basis, prokaryotic rRNA genes dominated in both size fractions regardless of the sampling depth.

### The contrasting diversity patterns and temporal stability between prokaryotic and protistan communities

Overall, prokaryotes and protists displayed opposite vertical diversity patterns (Fig. 3a); prokaryotic diversity increased with depth, whereas protists diversity decreased with depth. This diversity trends may be due to the differences in metabolic capacities between prokaryotes and protists. There is a persistent hypoxic environment below 500 m due to restricted circulation within the basin at SPOT [10, 11, 26, 27]. Unlike prokaryotes, which are capable of anaerobic and microaerobic metabolism, only a subset of eukaryotic taxa can adapt to oxygen-depleted layers. Previous studies from marine oxygen minimum zones (OMZ) have showed that protistan diversity decreases from surface waters to hypoxic waters [28, 29], which is consistent with our findings, supporting the importance of dissolved oxygen in shaping protistan communities. In addition, the high protist diversity at the surface was partly contributed by phototrophic or mixotrophic protists, which primarily occurred in the euphotic zone.

Temporal variability of pairwise Bray–Curtis similarity (a way to examine temporal stability) shows that protistan communities change considerably more over time compared with prokaryotic communities (mean Bray-Curtis similarity of each depth is 0.38–0.64 for prokaryotes vs. 0.12–0.19 for protistan). Moreover, Bray-Curtis similarity patterns of free-living prokaryotes at 5 and 890 m are essentially flat (Fig. 4a), indicating that the average relative proportions of all community members have stayed about the same for the entire 14 years, because imports, local extinctions, or any directional changes in composition over time would result in a decreasing similarity with increasing intervals, such as free-living prokaryotic communities at 150 m (Fig. 4a). The differences in temporal stability suggest there are much stronger factors stabilizing prokaryotic communities than protistan ones. The factors that influence the prokaryotic community stability potentially include large population size, diverse metabolic capacity, and high dispersal potential compared to protists. According to metacommunity theory, these characteristics can make prokaryotes relatively resilient to changing environments since they can survive or sustain a small population in unfavored environments [30].

We identified clear depth-differentiation within prokaryotic assemblages, explaining 48% of variability (Fig. 3c and Table 1), in line with previous reports [10, 31–38]. When considering seasonal changes by depth, the seasonal effect dramatically decreased below the euphotic zone (Table 2), suggesting seasonal drivers of prokaryotes are not major factors to the overall community in deeper waters. The protistan communities, on the other hand, were less influenced by depth, explaining 18% of variability (Fig. 3c and Table 1). When considering seasonal changes of protists by depth, the strongest seasonal effect was found unexpectedly at 500 m (Table 2), even though we recognize that season should affect protists more above the euphotic zone where phytoplankton respond to variations in light and nutrients. This finding suggests seasonal variations in transient sinking particles from surface waters may be large enough to affect overall diversity patterns in protistan communities (resident plus transient ones) at certain mid-depths.

### Spatiotemporal dynamics of prokaryotic communities in two size fractions

Throughout the water column, there is a strong vertical gradient in physicochemical variables (Fig. S1) as well as prokaryotic community composition (Fig. 3c and Table 1), in accordance with previously reported trends [1, 37, 39–42]. By sampling two size fractions in the water column, we found that free-living (0.2–1 μm) and particle-associated or larger (1–80 μm) prokaryotic communities exhibited significant differences in alpha- and beta-diversity, especially below the euphotic zone (Fig. 3 and Table 2). Particle-associated or larger prokaryotic communities exhibited a higher diversity than the free-living ones (Fig. 3a), which is consistent with previously studies [43–45]. We also found that dissimilarity between the two size fractions increased with depth (Table 2), suggesting that the progressive breakdown of sinking particles at depth may provide more ecological niches to be partitioned for particle-associated prokaryotes, compared to free-living prokaryotes subsisting on dissolved material.

Overall, the Shannon index of prokaryotes was lowest at 5 m (Fig. 4), consistent with previous findings that the alpha-diversity is lowest in the surface ocean [41, 42, 46]. In addition, the Shannon index was highest at 150 and 500 m. This appears attributable to the endemic taxonomic groups that were only present at depth (Fig. 6). These endemic groups were often more metabolically versatile groups that might be involved in nitrogen and sulfur cycles, as previously studies have found these taxonomic groups would mediate diverse redox reactions, such as Thiomicrospirales (sulfur oxidation), Marinimicrobia (nitrous oxide reduction), Nitrosopumilales (ammonia oxidation), Nitrospinales (nitrite oxidation), and SAR324 (sulfur oxidation) [47–50]. These profiles likely indicated a combined effect of different depth-varying physicochemical components including light intensity, organic matter composition, oxygen levels, redox substrates, and nutrients, as well as biological interactions between microbes.

In addition, vertical niche differentiation within SAR11 and Flavobacteriales was also found in the water column (Fig. 6b, c). SAR11 has been classified into clades and subclades, previously reported to have restricted vertical distributions. For example, SAR11 clade Ia and Ib are associated with surface ocean, while SAR11 clade IIa is associated with the OMZ [20–24]. Consistent with some previous findings, we found SAR11 clade II present in high ASV diversity in both size fractions below the euphotic zone, especially at 150 and 500 m. However, SAR11 members are widely believed to be free-living bacteria growing on mostly small molecules in dissolved material, and significant enrichment on particles as we observed has not been discussed before. Our finding suggests that SAR11 clade II might have a previously unknown niche on particles. Flavobacteriales also displayed distinct vertical patterns, with diversity of Flavobacteriaceae peaking at surface, whereas diversity of NS9 marine group increased with depth. Flavobacteriales are highly specialized in the degradation of complex organic compounds [51]. To our knowledge, depth-differentiation within Flavobacteriales as we report has not been discussed before. Our results suggest niche-partitioning of Flavobacteriales along the water column.

Seasonal changes were particularly dominant in the euphotic zone (Fig. 5 and Table 2), where light and temperature have substantial seasonal variation (Fig. 1 and Fig. S2). Many of these abundance patterns likely resulted from heterotrophic response to variations in primary production, such as the annual peaks in spring (Fig. 1), often in blooms [4, 52–54]. Flavobacteriales, Rhodobacterales, and Cellvibrionales that showed abundance maxima during the spring blooms are known to be bloom-associated groups that are capable of degrading phytoplankton-derived polysaccharides [54], whereas oligotrophic groups with streamlined genomes such as SAR11 appear to dominate the euphotic zone the rest of time when nutrient conditions are low [4, 55]. In addition, seasonal effects greatly decreased below the euphotic zone (Table 2), suggesting that the seasonal downward transport of large, fast-sinking particles from the surface did not lead to strong seasonality of the deep community as a whole, even though several particular taxa have been shown to respond to sinking material at this site [10, 56]. This may suggest the overall community variation over time in the deep water is significantly affected by other factors besides seasonal sinking material.

The effects of size fraction on community composition, on the other hand, were more prominent below the euphotic zone (Table 2). The difference between the two size fractions resulted from many taxonomic groups that were enriched in either free-living or particle-associated size fractions (Fig. S9). We observed that members of Alphaproteobacteria (SAR11, Puniceispirillales, and Rhodobacterales) and SAR86 were enriched in the small size fraction in the euphotic zone, whereas Nitrospinales, Thiomicrospirales, SAR324, Marinimicrobia, Nitrosopumilales Archaea, and Marine Group II Archaea were enriched in the small size fraction below the euphotic zone. In addition, Actinomarinales and members of the Bacteroidetes (including Flavobacteriales), Planctomycetes, Verrucomicrobia, Betaproteobacteria, Gammaproteobacteria, and Deltaproteobacteria were enriched in the large size fraction. These patterns were mostly consistent with previously findings [43–45, 57–59], except for Actinomarinales, which has been previously found via single amplified genomes (SAGs) to be one of the smallest planktonic prokaryotes [60]; this apparent discrepancy might be explained if some abundant Actinomarinales are particle-associated, in contrast with SAGs that come from individual sorted cells.

### Spatiotemporal dynamics within protistan communities

We found depth changes along the water column in protistan assemblages (Figs. 3 and 8), which is consistent with the results obtained from previous studies [61–64]. As expected, photosynthetic groups (e.g., Prymnesiophyceae, Mamiellophyceae, and Bacillariophyta) dominated the euphotic zone, whereas heterotrophic groups, notably including Radiolaria (Polycystinea, Acantharea, and RAD-B), increased in relative abundance with depth [65, 66]. In addition, Syndiniales (previously called MALV-Marine Alveolates) were prevalent throughout the water column. Syndiniales have been characterized as parasites on a wide range of hosts, such as dinoflagellates, ciliates, and Radiolaria [67–69]. As reported by other studies of protists at SPOT and elsewhere [27, 63, 70–74], the widespread distribution of Syndiniales suggests the importance of parasitism throughout the water column.

We found that sampling season explained 6–9% of total variation at most depths, except for 500 m, where variation explained by season is two times stronger (14%). These findings are different from a previous study of protists at SPOT. Kim et al. [27] documented the seasonality of protistan communities between 2000–2003 at SPOT using terminal restriction fragment length polymorphism (T-RFLP), and they found seasonality at 150 m but not at 500 m. Due to the difference of methodology (T-RFLP vs. tag sequencing) and sampling period (2000–2003 vs. 2005–2018), the seasonality we observed at 500 m may not have been present or detectable in their study. However, both results showed how seasonal inputs of sinking materials from surface water can affect patterns of protistan assemblages at intermediate depths.

### Positive diversity relationships among groups

We found positive diversity relationships between protists/phototrophic eukaryotes and particle-associated prokaryotes. Given there are many potential microbial interactions among prokaryotes and protists, based on predation, parasitism, nutrient-sourcing, and resource exchange (including symbioses), the strong positive diversity relationships may be due to these microbial interactions. By analogy, empirical studies have found that diversities of prey and predator are often positively correlated with each other in marine and terrestrial ecosystems [75–80]. Such positive diversity relationships may result from three different mechanisms [1]: predator and prey are driven by the same environmental forces and thus positively correlated [2] higher prey diversity can promote predator diversity by providing diverse resources, [3] higher predator diversity can suppress dominant prey and thus prevent competitive exclusion, resulting in higher prey diversity. Mechanisms [1] and [2] would apply to prokaryotes and eukaryotes interacting in all the ways mentioned above. Mechanism [3] would apply to protistan predators and prokaryotic prey. Interestingly, no correlation was found between eukaryotes and free-living prokaryotes, suggesting that physical associations yield much stronger interactions between specific prokaryotes and protists than do interactions at a distance via dissolved materials.

## Conclusions

In this 14-year long-term study, we quantitatively measured the proportion of prokaryotic 16S, chloroplast 16S, and 18S rRNA genes all at the same time using universal primers, and found different diversity patterns among protists, free-living, and particle-associated or larger prokaryotes. Based on these patterns, we found significant differences in long-term stability between prokaryotes and eukaryotes: free-living and particle-associated prokaryotic communities largely persisted over time, whereas eukaryotic communities changed much more dramatically at the ASV level. All these different patterns may be due to underlying causes that include differences in population sizes, abilities to adapt to oxygen-depleted environments, predation/parasitism/virus infection, and trophic status. All of these need further investigation. This study can serve as a baseline for monitoring spatiotemporal dynamics of whole microbial communities, facilitated by their analysis via a single PCR reaction, resulting in relative abundances with a shared denominator. Putting all organisms into the same quantitative context can show us shifts in the relative importance of prokaryotes and eukaryotes and helps us better follow and understand their long-term trajectories and responses to environmental changes.

## Materials and methods

### Sample collection and DNA extraction

Samples were collected monthly at the SPOT location from 5 depths, including 5 m, chlorophyll maximum depth (DCM), 150, 500, and 890 m, between 2005 and 2018. Ten to fifteen liters of seawater was sequentially filtered through an 80-μm mesh, a 1-μm A/E filter (Pall, Port Washington, NY), and a 0.2-μm Durapore filter (ED Millipore, Billerica, MA). Filters were stored at −80 °C until DNA extraction. Durapore filters (collecting material 0.2–1 μm) were used for free-living prokaryotic community analysis, and A/E filters (collecting material between 1–80 μm) were used to analyze phytoplankton, microzooplankton and particle-associated or larger prokaryotic communities. DNA was extracted from the Durapore filters using a hot SDS, phenol/chloroform/isoamyl alcohol, ethanol precipitation extraction protocol as described by Fuhrman et al. [79]. DNA on the A/E filters was extracted using a NaCl/CTAB bead-beating extraction protocol as described by Lie et al. [80] with slight modification by adding an ethanol precipitation step after lysis to reduce the volume of crude extract, which helps minimize DNA loss during the subsequent purification.

### PCR and sequencing

The V4-V5 hyper-variable region of the 16S and 18S rRNA genes were amplified simultaneously using a universal primer set 515Y (GTGYCAGCMGCCGCGGTAA) and 926R (CCGYCAATTYMTTTRAGTTT). All DNA samples were amplified and purified using the same conditions described at doi.org/10.17504/protocols.io.vb7e2rn [3]. Purified PCR products were pooled in equal amount and then sequenced on Illumina HiSeq 2500 in PE250 mode or MiSeq PE300. For each sequencing run, multiple blanks (i.e., PCR water) and four versions of mock communities (eukaryote and prokaryote, both even and staggered) were included as controls, meaning they were amplified, cleaned, and sequenced as environmental samples with the same conditions. This way, results from different sequencing runs were comparable without significant instrument bias and contamination [5, 81].

### Sequence analysis

The forward and reverse sequences were submitted to the EMBL database under accession number PRJEB48162 and PRJEB35673. Scripts necessary to reproduce the following analysis are available at github.com/jcmcnch/eASV-pipeline-for-515Y-926R. Briefly, amplicon sequences were trimmed with cutadapt, discarding any sequence pairs not containing the forward or reverse primer (error rate = 0.2). Amplicon sequences were then split into 16S and 18S pools using bbsplit.sh from the bbtools package (http://sourceforge.net/projects/bbmap/) against curated 16S/18S databases derived from SILVA 132 [82] and PR2 [83]. The 16S and 18S amplicons were then analyzed in parallel to ASVs using DADA2 [12] implemented in QIIME2 [84]. For 16S analysis, forward and reverse 16S reads were truncated (at positions where the quality distribution drops below 25), denoised, merged through their overlaps, and filtered for chimeras using dada2 denoise-paired commands. 16S ASVs were then classified with qiime2 classify-sklearn plugin against the SILVA 132 database [82]. 16S ASVs identified as Mitochondria were removed. Then, the ASV table was subdivided into prokaryotic 16S ASV table and Chloroplast 16S ASV table (including all 16S ASV identified as Chloroplast). Chloroplast 16S ASVs were further classified against PhytoRef database [85]. 18S analysis was different because 18S amplicons from 515Y/926R are too long (575–595 bp) for forward and reverse reads to overlap using current MiSeq and HiSeq platforms (so would be lost from our 16S pipeline that requires overlaps). Therefore, forward and reverse 18S reads were trimmed to 220 bp and 200 bp respectively using BBduk from bbtools and concatenated using fuse.sh from bbtools. Then the concatenated 18S reads were denoised and filtered for chimeras using dada2 denoise-single commands. This sequence processing strategy has been validated with 18S mock communities [3]. 18S ASVs were assigned against the PR2 databases [83].

### Environmental data

Variables including temperature, oxygen, and fluorescence were recorded by a CTD. Nutrient variables including nitrite, nitrate, and phosphate were analyzed by MSI Analytical Lab at UCSB. Satellite sea surface temperature, chlorophyll-a concentration and surface productivity estimates were download from the Coastwatch browser website.

### Sequencing bias correction

The 515Y/926R amplification has been tested with 16S and 18S mock communities mixed in equimolar quantities, and results have shown that there is a two-fold underestimation of 18S sequences due to sequencing length bias [3]. Thus, a two-fold correction factor was used to estimate the true proportions of prokaryotic 16S, chloroplast 16S, and 18S in each sample, and these are used in the main results. The uncorrected proportions of the three categories are shown in Fig. S3.

### Statistical analysis

All statistical analyses and visualization were conducted with R (v4.1.0) using VEGAN [86], ggplot2 [87], pheatmap [88], and GGally [89]. Shannon index and Bray-Curtis distance were calculated using *diversity* and *vegdist* functions. NMDS was performed using *metaMDS*. The differences in the alpha-diversity between groups were evaluated with the Kruskal–Wallis test using *kruskal.test*, and the Dunn’s test was used as the post-hoc test using the *dunn.test*. The statistical differences in community composition among sampling depths, seasons, and size fractions were evaluated with PERMANOVA using *adonis2* function with 999 permutations. Since the interactions of different variables had less impact, explaining <5% the variations, interaction terms were not included in the final PERMANOVA model. Correlation analyses were performed using *ggpairs* function.

## Supplementary information

Supplementary information
