# Supplementary material for: Contrasting diversity patterns of prokaryotes and protists over time and depth at the San-Pedro Ocean Time series
Source: ISME Commun. 2022 Apr 13;2:36. doi: 10.1038/s43705-022-00121-8 (PMC9723720; doi:10.1038/s43705-022-00121-8)
Supplement: Supplementary file 1 — Supplementary information [file 43705_2022_121_MOESM1_ESM.pdf]

Supplementary information

Contrasting diversity patterns of prokaryotes and protists over time and depth at the San-Pedro Ocean Time-series

Yi-Chun Yeh[1] and Jed A. Fuhrman[1]

[1]Department of Biological Sciences, University of Southern California, Los Angeles, California 90089-0371, USA

Correspondence:

Jed A. Fuhrman

Department of Biological Sciences, University of Southern California, Los Angeles, California 90089-0371, USA

Email: fuhrman@usc.edu

Figure S1. Principal component analysis (PCA) of environmental variables measured between 2005-2018. The environmental variables analyzed are indicated as vectors: CTD temperature (temperature), CTD dissolved oxygen (oxygen), CTD chlorophyll fluorescence (fluorescence), nitrite and nitrate (NO_2+NO_3), and phosphate (PO_4). Samples are color-coded by sampling depth.

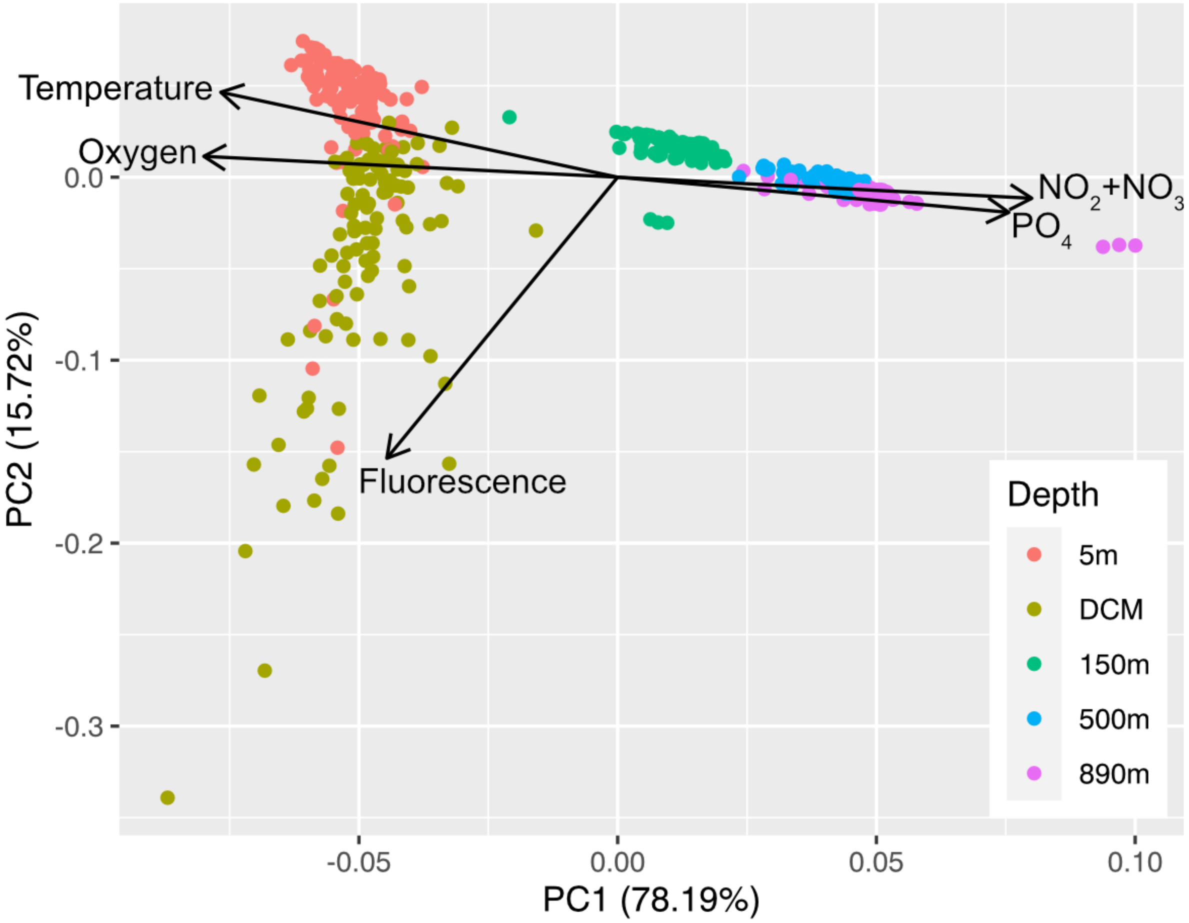

Figure S2. The temporal variation of physiochemical variables measured at different depths.

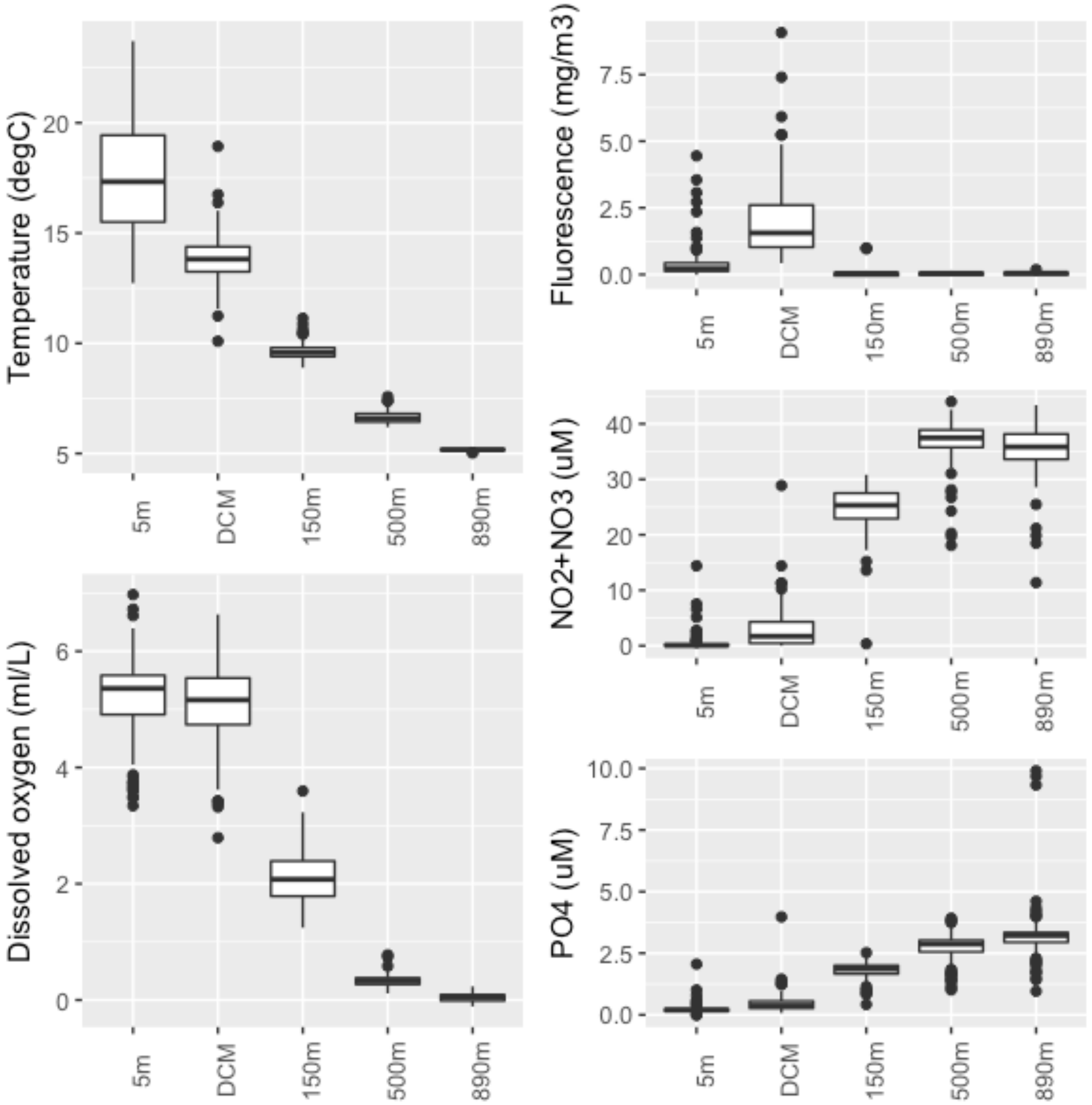

Figure S3. Dominance of prokaryotes in both size fractions, as shown by the proportions of 16S, chloroplast, and 18S reads (including Metazoa sequences) found in 0.2-1 µm and 1-80 µm size fractions. Note these values are uncorrected for the estimated 2-fold sequencing biases against 18S sequences compared to shorter 16S sequences (corrected values are in the main paper Fig. 2).

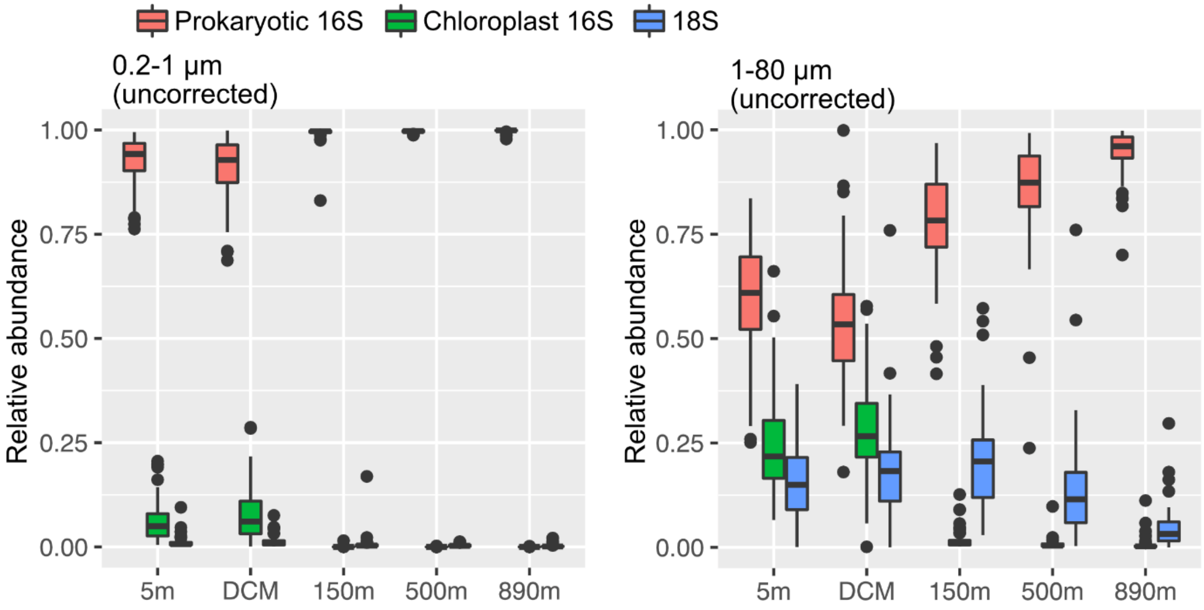

Figure S4. Temporal variation of free-living prokaryotic 16S, particle-associated or larger celled prokaryotic 16S, and 18S (excluding Metazoa sequences) communities by plotting Bray-Curtis similarity between all sampling months for each sampling depth. Free-living (0.2-1 µm) and particle-associated or large (1-80 µm) prokaryotes at 5 m and DCM exhibited a clear annually recurring pattern, with peaks at 12, 24, 36 etc. month intervals (same calendar months), and troughs at 6, 18, 30 etc. intervals (opposite seasons), whereas prokaryotes at depth exhibited a relatively steady community over time (larger Bray-Curtis similarity), especially in the small size fraction. Eukaryotic community composition, on the other hand, was relatively unstable over time compared to prokaryotes, as shown by much lower average similarities.

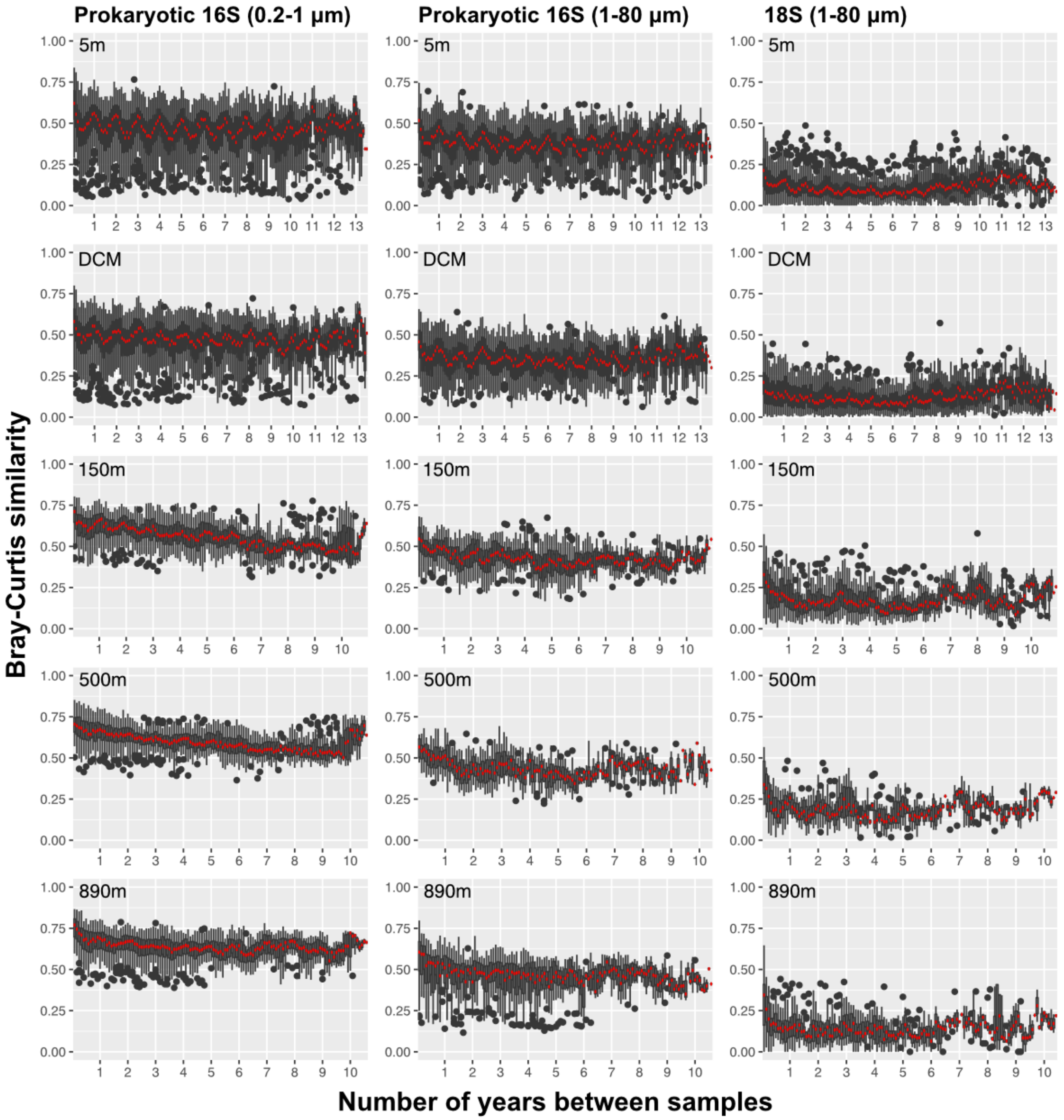

Figure S5. Temporal patterns of community similarity of chloroplast 16S by plotting the Bray-Curtis similarity between all pairs of sampling months within each sampling depth (i.e., 5m and DCM), comparable to Fig S4. These data show the eukaryotic phytoplankton communities had an intermediate extent of annually repeating composition and overall stability, between that of the prokaryotic communities (high) and the entire protist 18S communities (low).

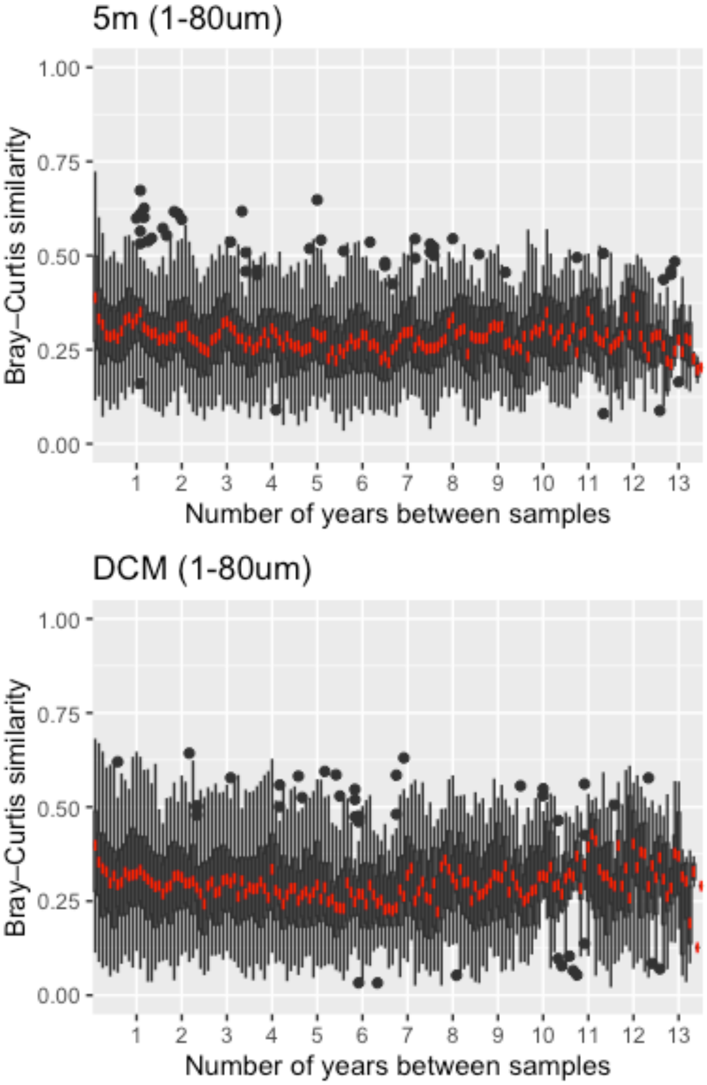

Figure S6. Heatmap of monthly average 18S communities (including Metazoa sequences) in the 1-80 μm size fraction at the class level (only dominant classes were selected, if their relative abundance is >2% of any samples). The columns were clustered based on Bray-Curtis distance. Rows were clustered based on Euclidean distance.

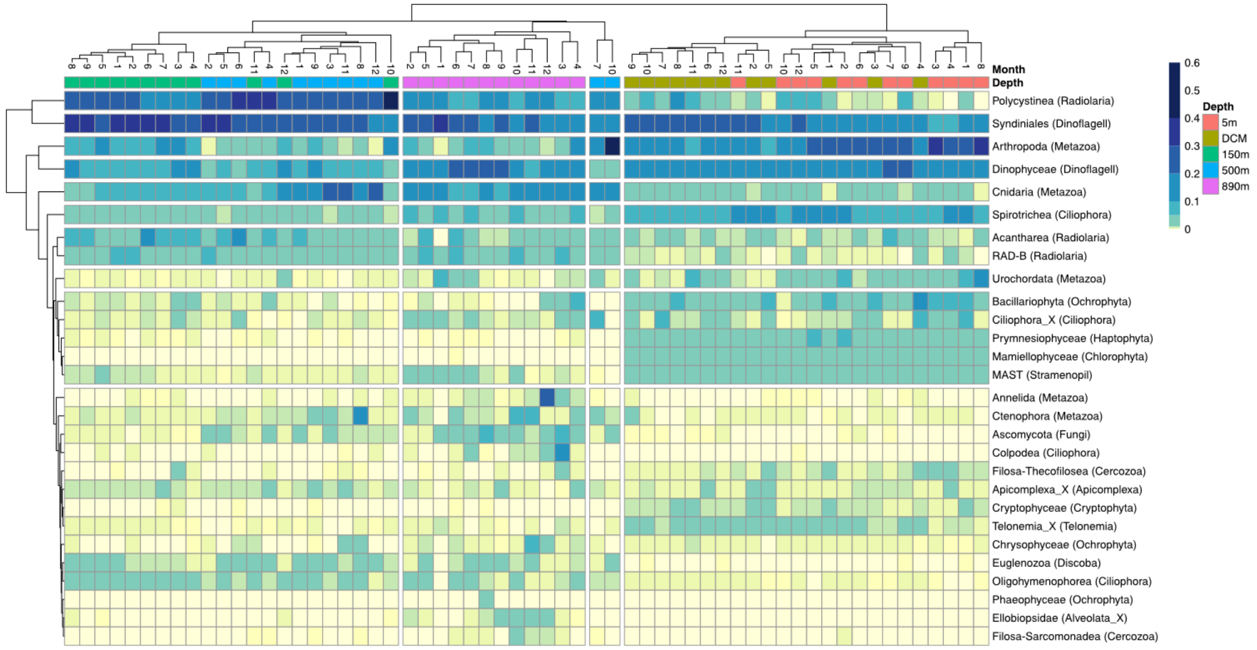

Figure S7. Heatmap of monthly average chloroplast 16S communities and phototrophic 18S communities at 5m and DCM in the 1-80 µm size fraction. For phototrophic eukaryotic 18S, we selected only ASVs corresponding to photosynthetic groups (divisions Chlorophyta, Cryptophyta, Haptophyta, Katablepharidophyta, Ochrophyta, Rhodophyta, Streptophyta and class Filosa-Chlorarachnea). Chloroplast 16S and phototrophic 18S communities both showed that phytoplankton were mostly dominated by Prymnesiophyceae in summer/autumn and Bacillariophyta in spring/winter

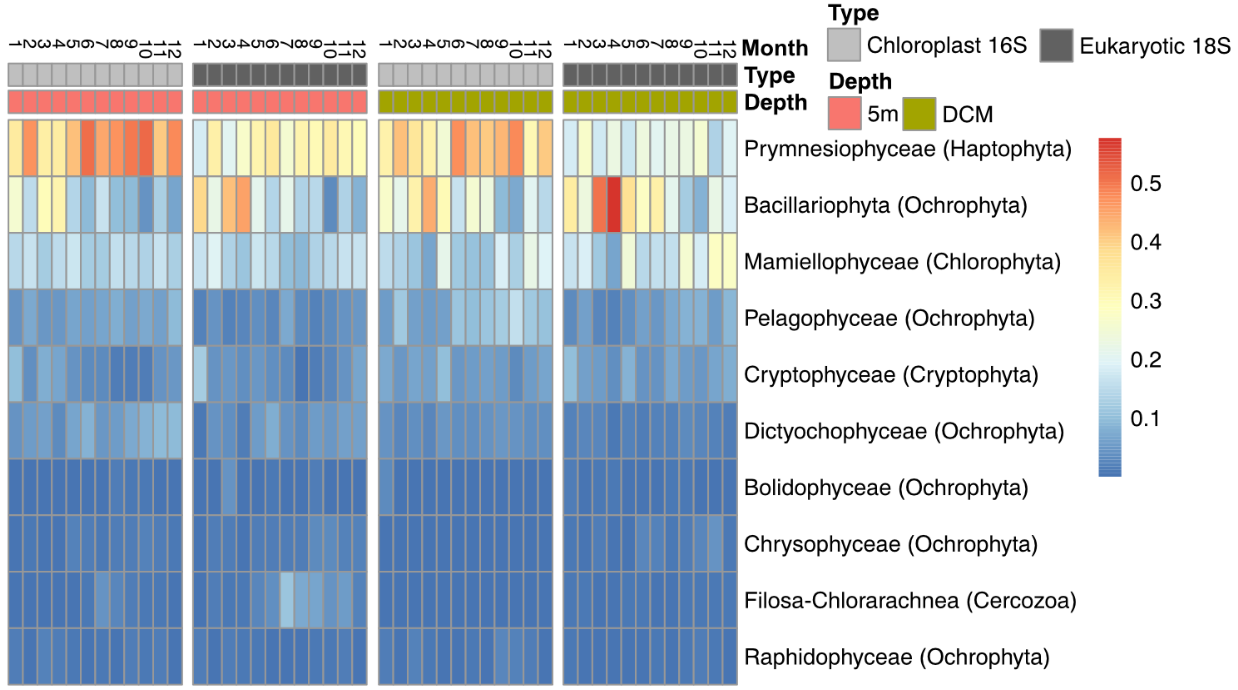

Figure S8. Heatmap of monthly average chloroplast 16S communities and phototrophic 18S communities in the 1-80 µm size fraction both showed that the phototrophic eukaryotes at 150m were mainly sinking diatoms (Bacillariophyta). For phototrophic eukaryotic 18S, we selected only ASVs corresponding to photosynthetic groups (divisions Chlorophyta, Cryptophyta, Haptophyta, Katablepharidophyta, Ochrophyta, Rhodophyta, Streptophyta and class Filosa-Chlorarachnea).

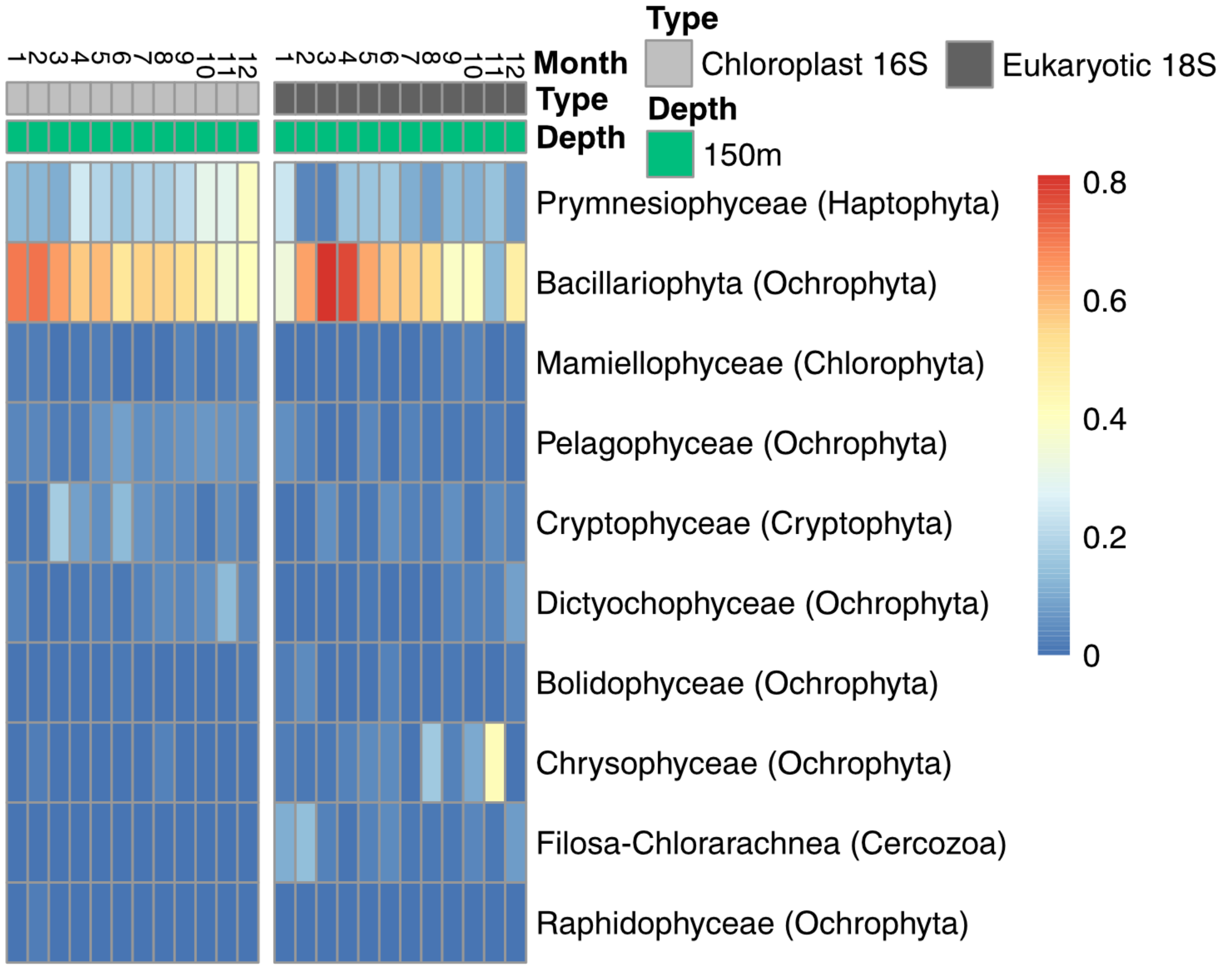

Figure S9. The heatmap of depth average prokaryotic communities, scaled and normalized within rows to examine how each taxon was distributed among depths and size fractions. Note this does not show relative abundances between taxa.

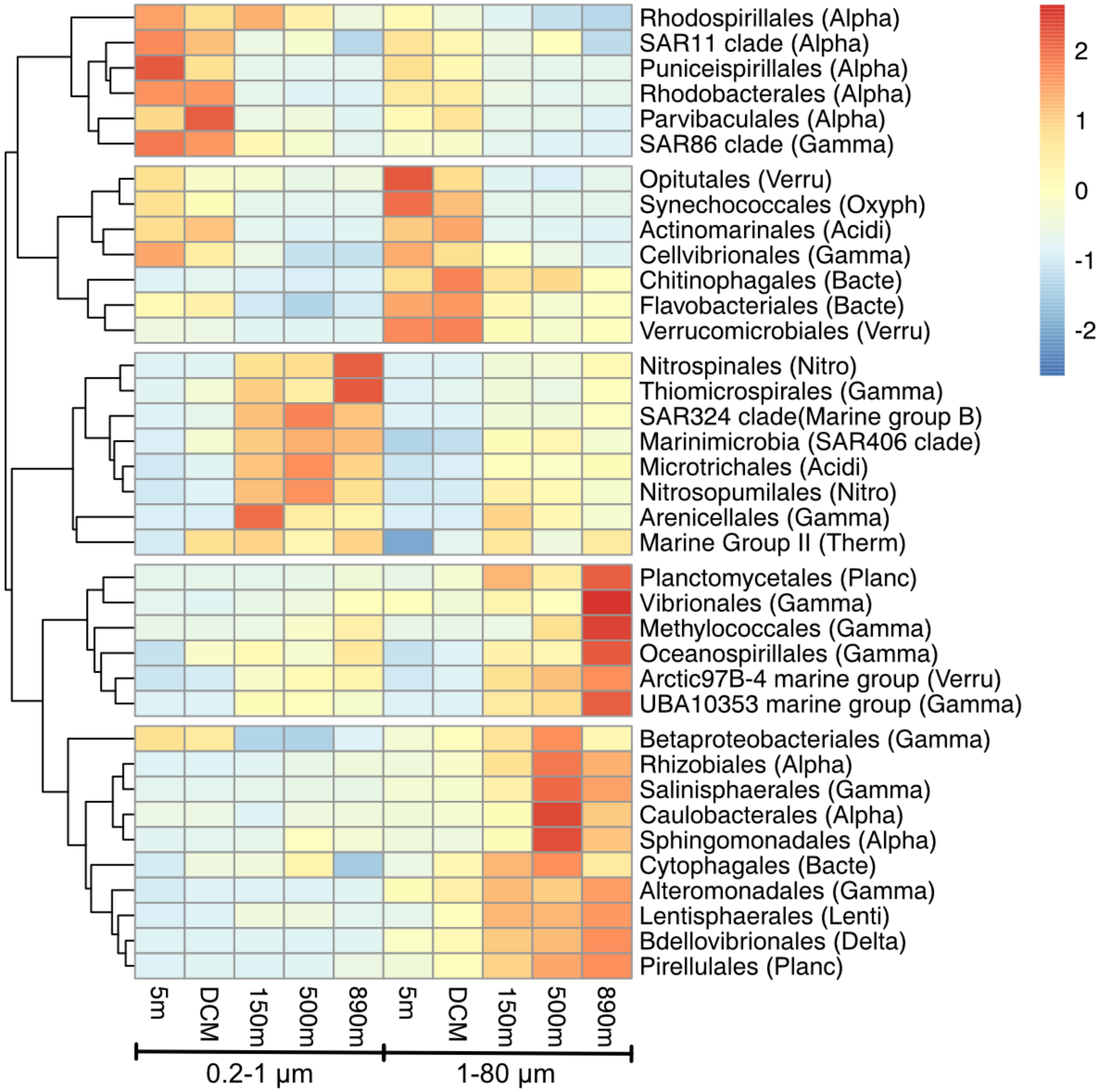

Figure S10. The comparison of relative abundance of chloroplast 16S vs. phototrophic eukaryotic 18S in the 1-80 µm size fraction, i.e. correspondence of 16S chloroplast and 18S based estimates of eukaryotic phytoplankton abundance. For phototrophic eukaryotic 18S, we selected only ASVs corresponding to photosynthetic groups (divisions Chlorophyta, Cryptophyta, Haptophyta, Katablepharidophyta, Ochrophyta, Rhodophyta, Streptophyta and class Filosa-Chlorarachnea). There were 22 classes detected in both chloroplast 16S and eukaryotic 18S communities. Blue lines are linear regression line, and black lines are 1:1.

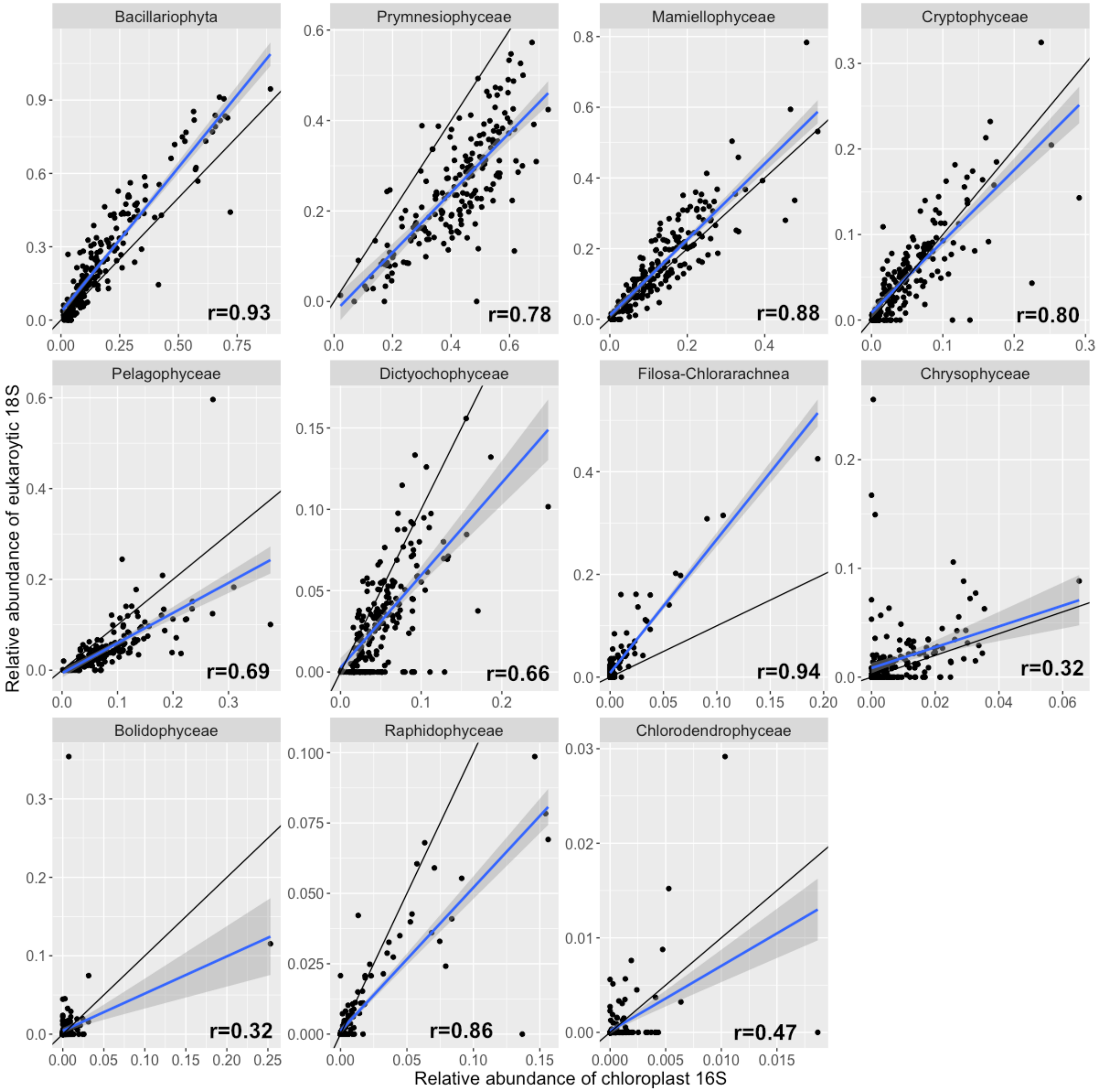

Figure S10. (continued)

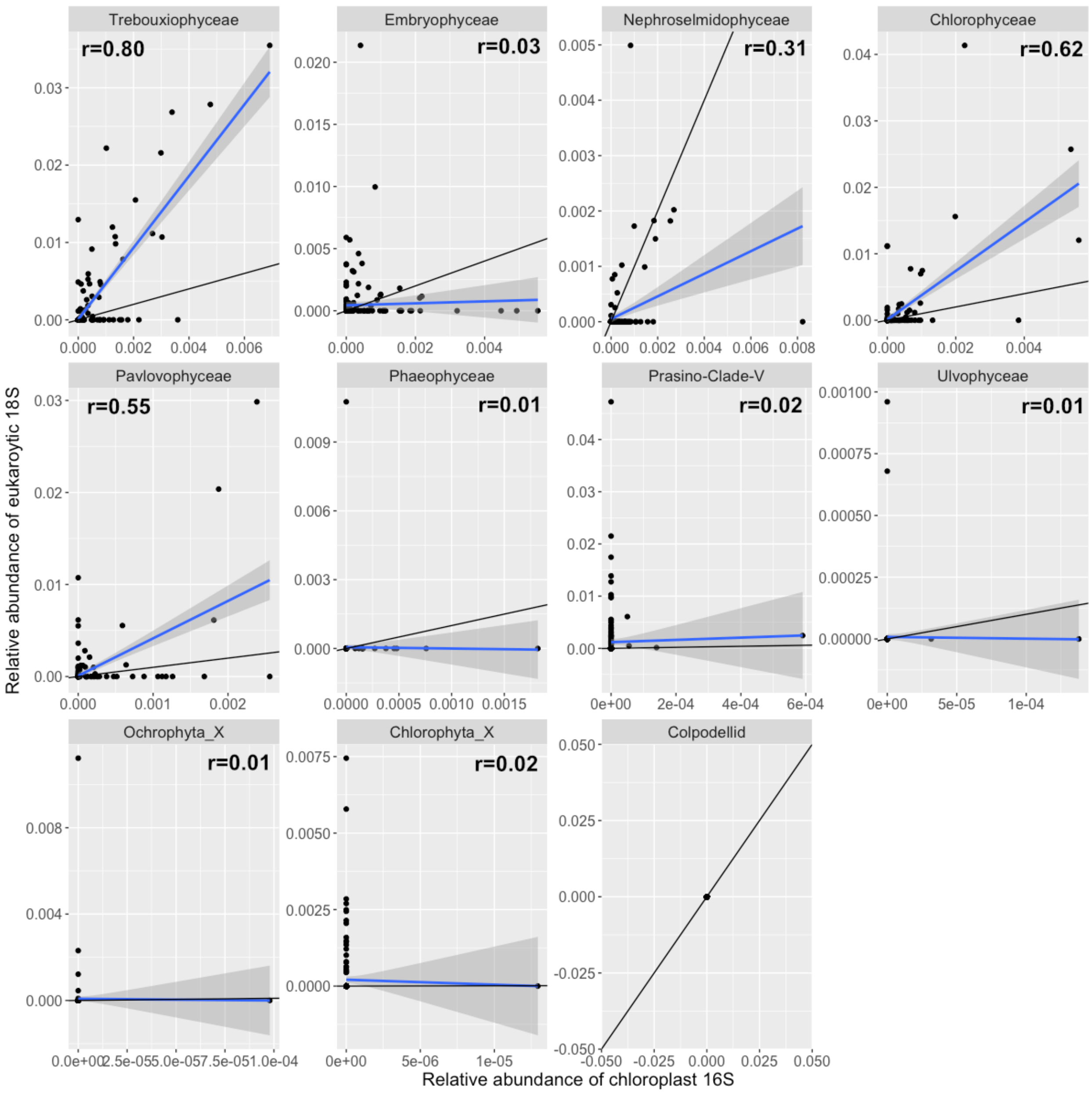

Figure S11. 8 classes that were only detected in chloroplast 16S communities in the 1-80 µm size fraction, i.e. no corresponding 18S sequence with the same taxonomy was found (potential database imperfection such as different taxonomies or missing sequences between databases, or aberrant sequences)

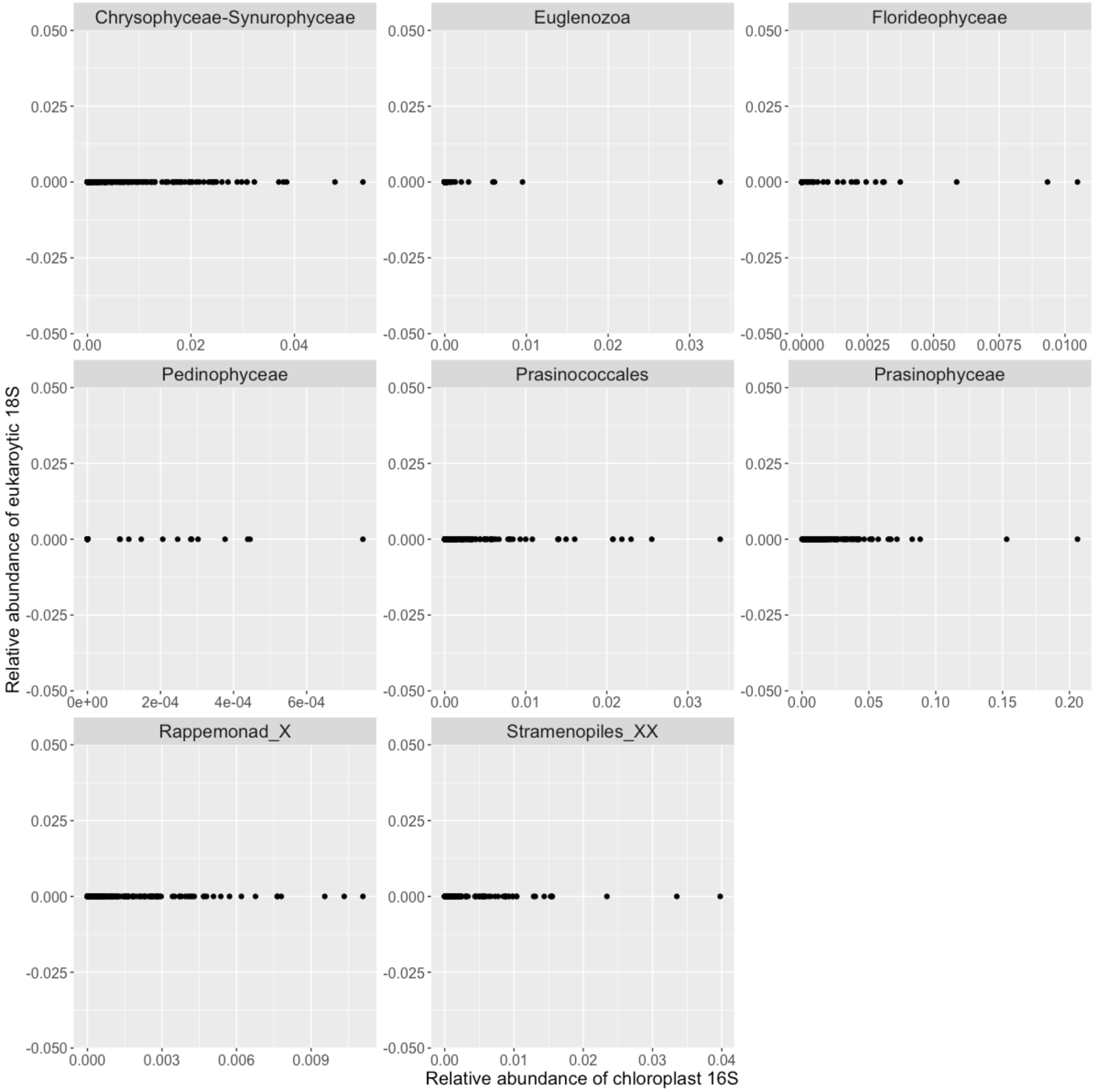

Figure S12. 13 classes that were only detected in phototrophic eukaryotic 18S communities in the 1-80 µm size fraction, i.e. no corresponding 16S chloroplast with the same taxonomy was found (potential database imperfection such as different taxonomies or missing sequences between databases, or aberrant sequences)

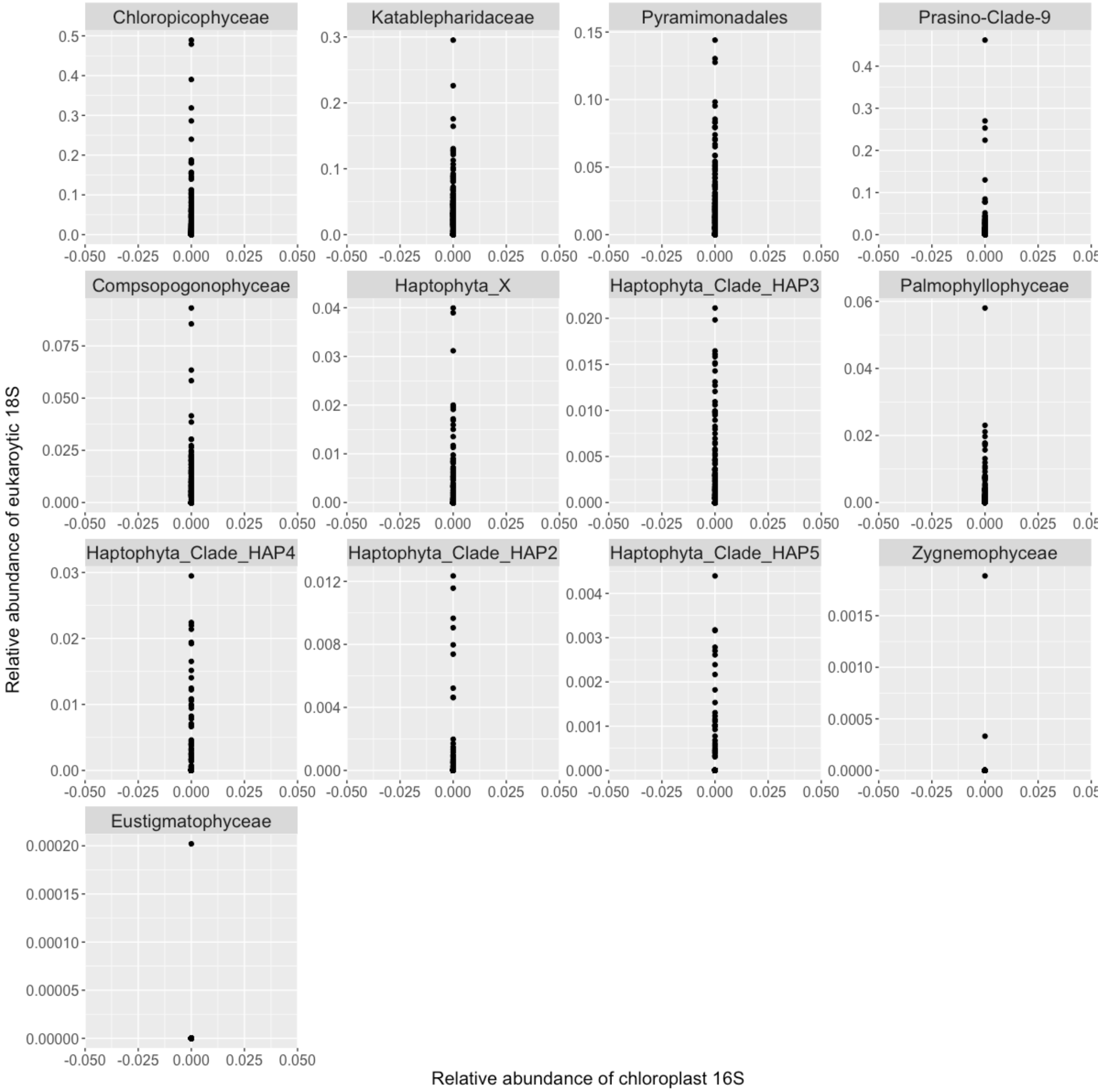

Phytoplankton communities using chloroplast 16S vs. eukaryotic 18S rRNA genes

As phototrophic eukaryotes in this study were examined by two marker genes, chloroplast 16S and eukaryotic 18S rRNA genes, the phytoplankton community composition (excluding dinoflagellates) analyzed with two marker genes were compared (Fig. S4). The results showed that seasonal dynamics at 5 m and DCM were similar and consistent with previous observations such that Bacillariophyta (diatoms) dominated during spring blooms, whereas the abundance of Prymnesiophyceae (haptophytes, mostly small flagellates) peaked in summer (78). Moreover, the relative abundances of the major groups were quite comparable when using chloroplast 16S and 18S rRNA genes (Fig. S7). For example, relative abundances of Mamiellophyceae and Cryptophyceae from chloroplast 16S and 18S fall close to the one-to-one lines. Prymnesiophyceae, however, were more represented when using chloroplast 16S. Bacillariophyta were slightly more represented when using eukaryotic 18S. This difference could be linked to variations in number of gene copies per genome (4, 79, 80) and number of chloroplasts per cell (81). Note that 18S rRNA gene copy number varies from one to thousands per genome, which precludes cell abundance estimation from 18S rRNA genes, though some analyses suggest that copy number variation relates generally (but not particularly accurately) to biomass (6, 82). On the other hand, the copy number of chloroplast 16S rRNA is only 1 or 2 per chloroplast, while the number of chloroplast per cell can vary from one to hundreds, which is a function of cell size, taxonomy, and to some extent environmental factors (4). Also, dinoflagellates are not represented by chloroplast 16S rRNA because the plastid DNA is highly fragmented (mini circles), preventing amplification (83). There were a number of classes that were reported only from 18S and others only from chloroplasts (Figs S11 and S12); some of

these are probably due to database imperfections (incomplete data, or different taxonomies between databases), while others may be due to aberrant or non-amplifiable sequences.
